# QTLMAS 2009: simulated dataset

**DOI:** 10.1186/1753-6561-4-S1-S3

**Published:** 2010-03-31

**Authors:** Albart Coster, John W M Bastiaansen, Mario P L Calus, Chris Maliepaard, Marco C A M Bink

**Affiliations:** 1 Animal Breeding and Genomics Centre, Wageningen University, Wageningen, The Netherlands; 2 Animal Breeding and Genomics Centre, Wageningen UR Livestock Research, Lelystad, The Netherlands; 3 Plant Breeding, Wageningen University, Wageningen, The Netherlands; 4 Biometris, Plant Research International, Wageningen, The Netherlands

## Abstract

**Background:**

The simulation of the data for the QTLMAS 2009 Workshop is described. Objective was to simulate observations from a growth curve which was influenced by a number of QTL.

**Results:**

The data consisted of markers, phenotypes and pedigree. Genotypes of 453 markers, distributed over 5 chromosomes of 1 Morgan each, were simulated for 2,025 individuals. From those, 25 individuals were parents of the other 2,000 individuals. The 25 parents were genetically related. Phenotypes were simulated according to a logistic growth curve and were made available for 1,000 of the 2,000 offspring individuals. The logistic growth curve was specified by three parameters. Each parameter was influenced by six Quantitative Trait Loci (QTL), positioned at the five chromosomes. For each parameter, one QTL had a large effect and five QTL had small effects. Variance of large QTL was five times the variance of small QTL. Simulated data was made available at http://www.qtlmas2009.wur.nl/UK/Dataset/.

## Background

In this article, we describe the simulation and the resulting data for the QTLMAS (Quantitative Trait Loci mapping and Marker Assisted Selection) Workshop 2009. An objective of the Workshop was to compare methods for detection of Quantitative Trait Loci (QTL) and methods for calculating breeding values with markers distributed over the whole genome, MEBV. The data represented measurements of a time-dependent trait, influenced by QTL, which could represent body mass of growing animals or biomass accumulated during growth of a crop.

The data consisted of phenotypes, biallelic single nucleotide polymorphism (SNP) genotypes, and family information. Phenotypes were simulated and made available for a subset of the simulated individuals at five different time points along the growth trajectory. SNP were distributed over the whole simulated genome. Some SNP were in linkage disequilibrium (LD) with QTL, but QTL information was not provided in the dataset. SNP genotypes were made available for the phenotyped and for the non phenotyped individuals. Simulated individuals were genetically related due to the small number of parents used. Data was made available at http://www.qtlmas2009.wur.nl/UK/Dataset/. The simulated dataset is available to be used as benchmark for methods that attempt to model QTL or breeding values related to growth functions.

## Simulation method

Simulated genomes consisted of five chromosomes of 1 Morgan each. At each Morgan, 10,000 loci were simulated. In the base population, allele frequency of 2,000 loci, equally distributed over the five chromosomes, was set at 0.5 and the remaining 48,000 loci were monomorphic in the base population. Fifty gametes were simulated for the base population according to these allele frequencies and these gametes were randomly combined into 25 genotypes.

Then, a thousand generations were simulated to create Linkage Disequilibrium (LD) between loci and to achieve a situation of mutation drift equilibrium. Two meioses were simulated for each genotype in a previous generation to maintain an effective population size of 49 [1]. Genotypes for the next generation were formed by combining random pairs of these gametes, while avoiding selfing.

The total number of recombinations in each meiosis event was drawn from a Poisson(5) distribution and recombination positions were distributed over the whole genome without interference. Throughout the 1,000 generations, mutation rate for all loci was 1 · 10^-5^, regardless of whether a mutation had occurred previously at this locus or not. A mutation did always reverse the allele: mutation of a 1 allele produced a 0 allele and mutation of a 0 allele produced a 1 allele.

**Figure 1:**
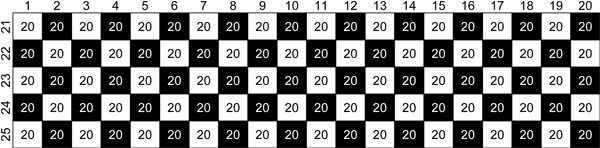
**Graphical representation of simulated generation 1,002.** Cell *i,j* represent the full sib family simulated by mating female *i* to male *j*. Black cells represent full sib families of which phenotype data was simulated; white cells represent full sib families of which phenotype data was simulated. Each full sib family consisted of 20 individuals.

Gametes in generation 1,001 were combined into 25 genotypes (combinations were random, selfing was avoided). From these 25 genotypes, 20 genotypes were regarded as *females* and 5 genotypes were regarded as *males*. For generation 1,002, a hundred full sib families were simulated by combining each female with each male. Each full sib family consisted of 20 offspring in generation 1,002. Figure 1 is a graphical representation of the mating structure used for simulating this last generation. Mutation rate was set at 0 during simulation of this last generation to avoid a large number of markers with very low Minor Allele Frequency (MAF).

Eighteen QTL were assigned to loci with MAF above 0.10 in generation 1,001. Additive effects for each QTL were calculated according to the variance required for that QTL and the allele frequency of that locus [2]. Variance of three QTL was set five times larger than variance of the remaining 15 QTL. Loci polymorphic in generation 1,001 to which no QTL was assigned became biallelic markers.

**Figure 2:**
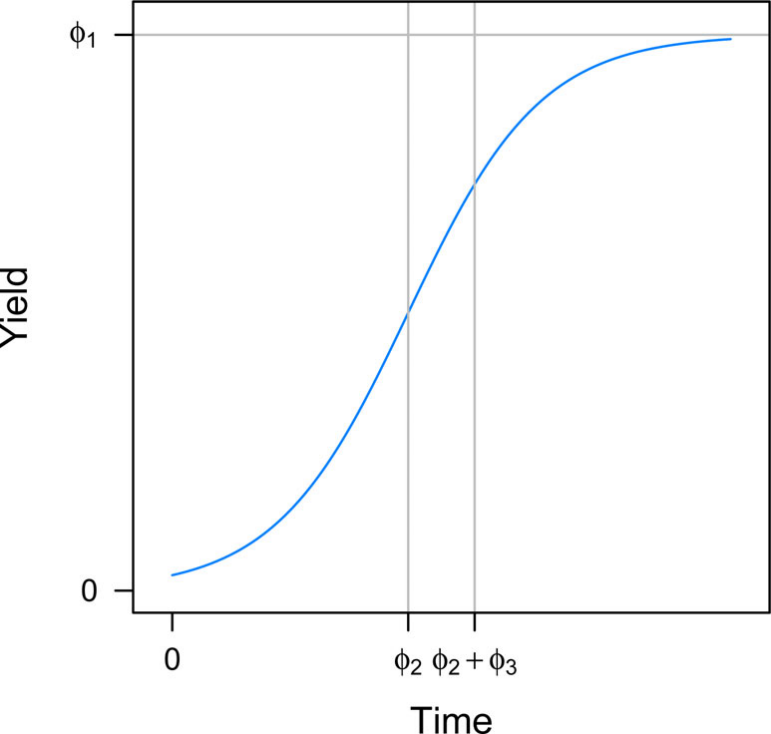
**Logistic growth curve.** Horizontal is the asymptote of the yield, represented with parameter *Ø*_1_ in the logistic function. First vertical line, at time = *Ø*_2_ is the inflection point. Second vertical line, at time = *Ø*_2_ + *Ø*_3_ is the point where the yield is approximately 0.83 of the asymptotic yield.

The simulated phenotype was yield (*y*), measured at five moments (*t*). Yield could represent weight during the growth of an animal or biomass during the growth of a crop. Yield at time t, *y(t),* was simulated according to a logistic growth curve:


				(1)

where *Ø*_1_ is the asymptotic yield, *Ø*_2_ is the inflection point of the curve and *Ø*_3_ is the slope of the curve. Figure 2 displays a growth curve and includes a graphical interpretation of the three parameters.

One large and five small QTL were assigned to each of the three *Ø* parameters. Simulated genetic value for each parameter was calculated as the sum of the additive effects of the QTL contributing to that parameter in each individual and  was the additive genetic variance of parameter *Ø*. Random normal deviates from a N(0,) distribution were added to genetic values of each parameter *Ø*. to simulate a heritability of 50%. Phenotypic observations at five moments in time were simulated and made available for participants of the Workshop. Phenotypes were calculated using the simulated phenotypic values for parameters *Ø*_1,2,3_ of each individual. A random normal deviate from a *N*(0,10^-4^*y_i_*(*t*)) distribution was added to observation *y_i_*(*t*) to simulate a small observation error.

Simulations were performed with HaploSim[3], a package for R [4]. 

## Simulation results

There were 453 polymorphic markers in the data, distributed over the genome. Average MAF of these polymorphic markers was 0.14 and average LD between flanking markers measured as r^2^ was 0.14. The fraction of QTL variance explained by the 10 markers in highest LD with each QTL was 0.83.

**Figure 3:**
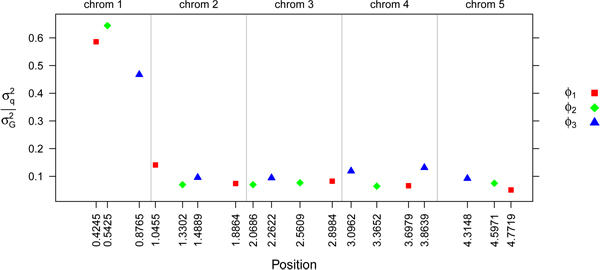
**Variance of QTL** for the three parameters at specific locations on the genome , expressed as fraction of the total genetic variance .

The three large QTL for parameters *Ø*_1,2,3,_ explaining approximately 50% of the additive genetic variance of each parameter, were located on chromosome 1. The remaining five small QTL for the parameters, explaining approximately 10% of the additive genetic variance of each parameter, were located on the other four chromosomes (Figure 3).

**Figure 4:**
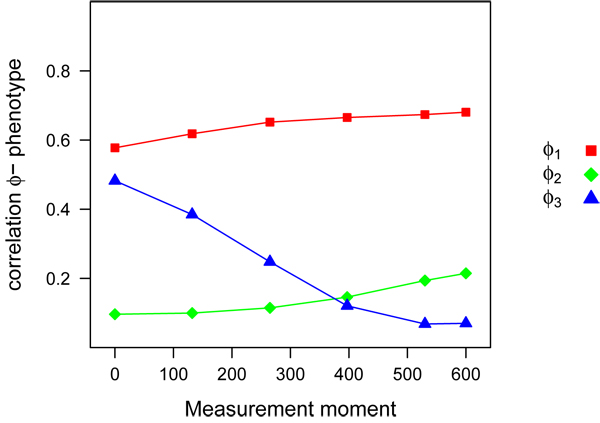
**Correlation between genetic value** for parameters* Ø*_1,2,3_ and phenotypes at the five measurement moments.

Simulated heritability of the parameters *Ø*_1,2,3_ was 0.50, implying a correlation of  between breeding values and the phenotypic parameter. Correlation between breeding values for the parameters and yield was lower than  and changed over time because yield was calculated with the logistic growth function, were the simulated parameters were used as parameters of the growth function (Figure 4). This has implications for the effectiveness of methods that attempt to estimate QTL or breeding values if the time dependency of the observations is not taken into account. Without taking the time dependency into account, the optimal moment for estimating QTL or breeding values for parameters *Ø*_1_ and *Ø*_2_ is at the end of the growing period whereas the optimal moment for estimating QTL or breeding values for parameter *Ø*_3_ is at the beginning of the growing period.

## Discussion

Objective of this simulation was to provide time-dependent phenotypes and marker data to the participants of the QTLMAS 2009 Workshop. Population size of 50 was used to fit with estimated population sizes of some common crop species. The density of polymorphic markers was much lower than currently assumed appropriate for genomic selection purposes [5,6], and was aimed not to impede other types of analysis. Simulations were performed in R using package HaploSim. Integration of simulations in a computation environment as R facilitates evaluation of simulation results using graphical and statistical functions provided in the environment. Programming simulations in using package HaploSim is relatively straightforward and the programmer has a high level of control over simulation results. Gametes were the basic simulation unit in this simulation because of the design of HaploSim.

With the objective to achieve a mutation drift equilibrium in relatively few generations of random mating, allele frequency of 2,000 loci was set at 0.5 in the base population. The number of generations of random mating required before reaching mutation drift equilibrium would be importantly higher if all loci were monomorphic in the base generation. On the other hand, setting allele frequency of all loci at 0.5 was not practical because computation time of HaploSim increases with the number of polymorphic loci.

Mutation rate was 1 ·  10^-5^ throughout the generations of random mating and this was justified as follows. In our simulations, loci correspond to base pairs. As an approximation, one centimorgan corresponds to 1 · 10^6^ base pairs [7]; one locus in our simulations thus corresponds to 1 · 10^4^ base pairs. Estimates for mutation rates in human are in the range 1 · 10^-8^ - 1 · 10^-7^[8,9]. Mutation rate in our simulations was therefore a factor 10 to 100 lower than base pair mutation rate estimated in human, with the objective to maintain a relatively low number of heterozygous SNP loci (equation 7.2.4, page 323, [1]). Mutation rate was set to 0 after generation 1,001 to avoid a large number of SNP with MAF equal to 1/4,000 (4,000 haplotypes were simulated for generation 1,002).

Phenotype data were calculated according to a logistic growth curve. A correct identification of this growth curve is expected to be crucial for successful identification of QTL involved in the simulated phenotypes. Conclusions about QTL position and QTL effect can be expected to be dependent upon this identification method [10]. This issue is further discussed in [11], in this issue.

Only additive QTL effects were simulated because methodology for QTL analyses with response curves is still limited (e.g. [10,12-18]). Simulations could easily be extended to scenarios involving dominance or epistatic interactions, however the objective of this Workshop was to look at the impact of having a time dependent trait on QTL mapping and estimation of breeding values and we did not want to mix complications of variable nature.

## Remarks

The code used to simulate the data can be obtained from the authors. Package HaploSim can be downloaded from the repository of R packages CRAN, http://cran.r-project.org/package=HaploSim, following the usual method to install R packages.

## Competing interests

Authors declare no competing interests.

## Authors' contributions

AC programmed the simulation, performed the analyses and wrote the manuscript. AC and JB wrote the simulation package HaploSim. CM, JB, MB, and MC were involved in the design of the simulation, in the evaluations of the results and in critically commenting the manuscript.
